# Impact of globalization on the environment in major CO_2_-emitting countries: Evidence using bootstrap ARDL with a Fourier function

**DOI:** 10.3389/fpubh.2022.907403

**Published:** 2022-09-08

**Authors:** Cheng-Feng Wu, Tsangyao Chang, Tsung-Pao Wu, Kai-jun Leng, Meng-Chen Lin, Shian-Chang Huang

**Affiliations:** ^1^Research Center of Hubei Logistics Development, Hubei University of Economics, Wuhan, China; ^2^School of Business, Wuchang University of Technology, Wuhan, China; ^3^School of Business Administration, Hubei University of Economics, Wuhan, China; ^4^Department of Finance, Feng Chia University, Taichung, Taiwan; ^5^CTBC Business School, Tainan, Taiwan; ^6^School of Accounting and Finance, Beijing Institute of Technology, Beijing, China; ^7^Department of Business Administration, National Changhua University of Education, Changhua, Taiwan

**Keywords:** bootstrap ARDL, globalization, CO_2_ emissions, Fourier functional form, unknown break date, China, the United States (US), India

## Abstract

Alongside sustainable development as a major global aim, the contribution made by globalization to environmental issues has become crucial in recent decades. Prior studies have focused on how trade in globalization influences the environment. However, multiple economic, social, and political factors are also important, the integration of which needs to be considered in sustainable development. Sharp and smooth breaks in time series models are the consequence of real-world structures. Using the bootstrap autoregressive-distributed lag test with a Fourier function, the present study reexamined the nexus between globalization and the environment in China, the United States, and India. The empirical results indicate that in the United States, the nexus between globalization and the environment is cointegrated in the long-term. In the short term, globalization is improving the environment in the United States and India. However, in China, globalization is resulting in environmental degradation. This research will assist policymakers in developing comprehensive strategies for sustainable development.

## Introduction

Globalization refers to a set of economic, social, and political structures and processes deriving from intra- or inter-continental networks of people, the links between whom are mediated by national economies, cultures, technologies, and governance (1, 2). The process of globalization benefits economic growth by advancing economic, social, and political development within a country; however, globalization is also increasing CO_2_ emissions, resulting in climatic change and environmental degradation (3). Consequently, the nexus between globalization and the environment is generating increasing levels of concern. Understanding this long-term relationship is essential for decision-makers striving to achieve sustainable development. To pursue such development, environmental, social, and economic factors must be considered (4).

Directly and indirectly, the process of globalization positively or negatively impacts the environment in three distinct ways: the income effect, the technique effect, and the composition effect (5). In the income effect, globalization results in CO_2_ emissions as a consequence of increasingly open trade and foreign investment (6). In the technique effect, globalization assists countries in manufacturing eco-friendly products through the inflow of energy-efficient technologies, thereby reducing CO_2_ emissions (7). However, the development of information and communication technology (ICT) does not reduce the consumption of energy during operations (8); therefore, the greater the development of ICT, the higher the consumption of energy, which can result in more CO_2_ emissions being ejected into the atmosphere. In the composition effect, globalization induces various forms of production depending on the comparative advantages for countries (9). Consequently, strict regulations to protect the environment in developed countries encourages enterprises to move their production activities and operations to developing countries where environmental regulations are weaker (10). This leads to the pollution haven effect, whereby polluting industries move from developed to developing countries (11). The composition effect also influences economic activity and CO_2_ emissions, depending on the degree of pollution from agricultural, industrial, and services sectors within in a country. Economic activities generate fewer CO_2_ emissions as a result of shifting from energy-extensive sectors to technology-intensive sectors (12, 13). The fact that globalization is linked to different components of activities means that previous studies investigating the nexus of globalization and the environment have employed disparate approaches, leading to mixed results and diverse inferences.

Within globalization, the economic, social, and political aspects of activities, including the trade of goods and services (14, 15), the arrival and departure of international tourists (16), ground processing and handling of aircraft (17), and internet usage (18) can alter the environment. For example, the United States introduced protectionist policies which impeded globalization during tariff wars in 2018. In the same year, China implemented environmental policies as ecological civilization is enshrined in its constitution. In 2020, tourism activities were reduced due to the emergence of the COVID-19 pandemic. Recently, the State Council in China released a report revealing its official aim to build an additional 160 airports, anticipating that 400 airports would be operational before the year 2035 (19). In India, environmental protection has become a challenge a new Draft Environmental Impact Assessment notification was released by officials which revealed that public consultation in several projects was lacking (20). Regional conflicts can be linked to economic and political circumstances at either a regional or global level. Since 2014, a conflict between Ukraine and Russia has been taking place; the Russian invasion of Ukraine in 2022 has altered the economic and political circumstances of the European region and the wider world. Specifically, the conflict has impacted political decisions, such as military deployment and action by the North Atlantic Treaty Organization, the United States, and China (21). In addition, it has caused chaos in the global supply chain. Disruption to supply from upstream suppliers is also affecting the production of goods because the region supplies natural gas, metals, and raw materials for the metal and technology industries worldwide (22). The above discussion reveals three aspects of globalization impacting the environment, highlighting the importance of decisions or events in a specific country.

Following the rapid development of globalization in recent decades, numerous studies have found evidence for its impact on the environment. Dreher et al. (23) conducted one of the first research studies to investigate how globalization affects environmental outcomes. Since then, a stream of time-series studies has suggested that globalization will result in environmental degradation. For instance, Yurtkuran (24) found that economic globalization is one of the factors that explain the increase in environmental pollution. Applying the globalization index as a proxy variable to assess the impact on environmental outcomes, Aslam et al. (25) reported similar results. However, another body of studies has found that globalization benefits environmental performance. Applying the ARDL method, Shahbaz et al. (7) reported a negative effect of the globalization index on CO_2_ emissions in India. Using Chinese data from 1980 to 2017, Umar et al. (26) conducted a similar study to explore causal relations between the globalization index and CO_2_ emissions. They found that globalization itself promotes better environmental outcomes. In addition to time-series methods, panel studies have been conducted to investigate the globalization-environment link. For instance, Kalayci (27) used panel data from NAFTA countries to explore the relationship between globalization and CO_2_ emissions. Their results indicated that both economic globalization and openness of trade negatively impact CO_2_ emissions. In a panel study of South Asian economies, Wen et al. (28) applied the fully modified ordinary least square method and revealed that increasing globalization increases CO_2_ emissions. In an investigation of the globalization and ecological footprint nexus, Yilanci and Gorus (29) examined panel Fourier Toda-Yamamoto causality in fourteen samples from Middle Eastern and North African (MENA) countries. Their results indicated that in most countries, the nexus exists when cross-sectional dependence among panel members is considered. Like Yilanci and Gorus (29), the current study applies the Fourier approximation in its proposed model to address the fact that time-series data contains structural breaks whose numbers, location, or forms are unknown a priori. However, rather than focusing on MENA countries, we investigated the three principal CO_2_-emitting countries where cross-sectional dependence is not the main issue. In sum, to measure the nexus between globalization and the environment, previous studies have collected data on specific variables such as openness of trade and the traditional globalization index (1). However, such studies are vulnerable to various proxy variables (30). Moreover, because the measurement of globalization varies, the results in such studies sometimes differ. Globalization is a process that produces complex relations of mutual interdependence. Therefore, using trade openness (6) and the traditional globalization index (29) as measures of globalization may not fully reveal its characteristics as the proxy variables or weight of variables in the traditional globalization index are relatively subjective.

Therefore, to measure globalization, a pluralistic and multiscale definition is preferred. For instance, Gygli et al. (2) revised the traditional globalization index to include comprehensive metrics. This revision was based on the fact that they viewed the metrics of de jure as a prerequisite for the metrics of de facto. In other words, a comprehensive globalization index is composed of both policy aspects and outcome aspects (31). Quinn et al. (30) found that when financial openness is measured by different proxy variables, one of which is de jure and the other is de facto, the nexus of financial openness and economic growth in two models varies. Thus, when the measurement of globalization is not comprehensive, the relationships between globalization and the variables of interest can be arbitrary. Furthermore, to measure globalization over time, the aggregation of individual variables requires the application of time-varying weighting techniques to account for structural changes in the relevance of individual variables. Thus, when examining the relationships between variables, the pluralistic and multiscale characteristics of the new globalization index can help to avoid subjective results. In addition, globalization metrics with respect to society and economies may change gradually or sharply during the process of globalization. For example, taxes on international trade as metric of de jure in trade globalization underwent an acute change in the United States (US) in 2019 following the initiation of the China-US trade war in 2018. For another example, the number of McDonald's restaurants as a metric of de facto in a country will gradually change as the number of facilities opened is typically identified in the network design phase where a company determines the structure of its supply chain for the next few years (32). Political, economic, and social change may create different levels of magnitude in the metrics. Shehzad et al. (33) used the ARDL bounding approach to examine how the impact of several macroeconomics factors and economic globalization on the environment in Algeria. The results showed that long-term cointegration exists when environmental quality is taken as the dependent variable; also, a negative effect of economic globalization on environmental quality exists in the short term. Different from Shehzad et al. (33), our study considers possible political decisions, economics, and social change when examing the nexus of globalization and environmental quality. Smooth and sharp breaks are important and have been considered in previous studies due to the fact that the results of a model will be impacted by the effects of shocks on the metrics (34). If structural breaks in a model are not considered, the results may be biased and generate misleading conclusions (35, 36).

According to IEA (37), the global trend in CO_2_ emissions has increased in recent decades, although major/minor events and effects have contributed to making this either a sharp/gradual decrease. In 2020, global CO_2_ emissions decreased by almost 6 percent, the greatest ever reduction and an almost five-fold decrease compared to the global financial crisis in 2009. However, the evolution of global activities is recovering as the demand for coal, oil, and gas increases. Consequently, global energy-related CO_2_ emissions are expected to increase by nearly 5% in 2021. According to the World Bank, over half of global CO_2_ emissions in 2018 came from China, the United States, and India. China is the world's largest emitter of CO_2_ with more than ten million kilotons, while the United States and India are the second and third largest CO_2_ emitters with nearly five million kilotons and two and a half million kilotons, respectively. Thus, examining the CO_2_ emissions in these three major CO_2_ emissions countries is vital in the trend of globalization.

Although single-country and panel studies have examined the nexus between globalization and the environment, no consensus has yet been reached. Also, other studies have applied the traditional cointegration model but have drawn inconclusive inferences (38–40). Limited studies have considered the novel globalization index and unknown breaks when examining the nexus between globalization and the environment in specific countries. In light of the above motivations and against the backdrop of the prior studies, the present research aims to reexamine the effects of globalization on the environment, especially for the major CO_2_-emitting countries. To the best of our knowledge, this is the first study to investigate the relationship between globalization and the environment in major CO_2_-emitting countries using the bootstrap autoregressive-distributed lag test with a Fourier function. It contributes to the existing literature in the following ways. First, it employs a relatively novel globalization index to investigate the relationship between globalization and the environment. Secondly, the proposed model employs a Fourier function which captures structural breaks; the effect of which is usually ignored in causality and unit root tests (34, 41). A Fourier function considers multiple structural changes where the number, location, or forms are unknown a priori (42). Third, it focuses on three countries that are responsible for a substantial proportion of global CO_2_ emissions. Based on the results, it offers policy recommendations that will motivate governments to be concerned about globalization conditions and environmental sustainability, and to build an acceptable sustainable development plan to achieve human development goals. The remainder of this paper is organized as follows: Section Data and method explains the data and method employed. The results are presented in Section Results and discussion. Section Conclusion discusses the results and provides concluding comments.

## Data and method

In this study, the influence of globalization on the environment was assessed using time-series data from 1970 to 2018 in China, the United States, and India. The recently developed globalization index was employed as a proxy of globalization and CO_2_ emissions were used as a measure of the environment. The latter was measured in kilograms in terms of GDP based on the 2010 constant price. In prior research, the KOF globalization index was taken as the proxy variable to measure globalization in the United States (43), China (44), India (45), and a panel study (46). On the other hand, CO_2_ emissions were taken as the proxy variable to measure the environment in prior studies (47, 48). Thus, following prior studies, this study used the KOF globalization index as the proxy for globalization as well as CO_2_ emissions as the proxy for the environment. Regarding data sources, the globalization index and CO_2_ emissions were retrieved from KOF Swiss Economic Institute and the World Bank, respectively. The time series of the globalization index was processed using logarithms, as logarithmic transformation reduces the heterogeneous effect when variables have a dynamic range. Table 1 presents summary statistics of the variables. Jarque-Bera statistics indicate that globalization for these three countries is not normally distributed. Regarding CO_2_ emissions, the data in China is normally distributed while the reverse is the case for the United States and India.

**Table 1:** Summary statistics of the variables.

**Country**	**Variables**	**Mean**	**Max**	**Min**	**STD**	**Skewness**	**Kurtosis**	**J-B**
China	CO_2_	2.620	4.979	0.948	1.338	0.435	1.635	5.347^*^
	Globalization	3.698	4.163	3.061	0.385	−0.182	1.478	4.995^*^
The United States	CO_2_	0.539	0.909	0.276	0.186	0.478	2.149	3.345
	Globalization	4.261	4.410	4.075	0.112	−0.306	1.628	4.605^*^
India	CO_2_	1.038	1.203	0.861	0.087	−0.077	2.264	1.154
	Globalization	3.718	4.130	3.382	0.288	0.323	1.391	6.136^**^

* Represent significance level in 10%.

** Represent the levels of significance in 5%.

This study re-examined the long-run cointegration and causal relationship between globalization and the environment by applying bootstrap ARDL with a Fourier function. The ARDL model was initially developed by Pesaran et al. (49), based on which McNown et al. (50) proposed the bootstrap ARDL model. Recently, Yilanci et al. (39) developed the bootstrap ARDL model with the Fourier approximation. The advantages of the bootstrap ARDL model with the Fourier function over conventional cointegration tests are as follows. First, the bootstrap ARDL test, unlike the conventional ARDL bounded test, allows for (weak) endogeneity of two or more variables of interest, which is valuable for handling feedback from the dependent to the independent variable. Secondly, in building suitable power and size properties, the bootstrap test is more rigorous than the asymptotic test when testing the conditions for cointegration. Thirdly, a lagged independent variable is incorporated into the model such that an additional test is added to support the F and t cointegration tests suggested by Pesaran et al. (49). Finally, the Fourier function technique overcomes the drawbacks of traditional tests for cointegration by allowing structural breaks whose numbers, location, or forms are unknown a priori. In the bootstrap ARDL method, the integration order for the variables of interest is I (0) or I (1); thus both stationary or non-stationary time series are available for use in examining cointegration (50).

To explore the cointegration between globalization and CO_2_ emissions, the following model is proposed.


(1) $$\begin{array}{l} y_{t}=\text{c}+\beta x_{t}+\varepsilon \text{t} \end{array}$$

where c is a constant, y represents CO_2_ emissions, and x denotes the globalization index.

Drawing on the conceptual framework of prior studies, the relationship described in Equation (1) can be tested using the ARDL bootstrap test developed by McNown et al. (50). For this purpose, Equation (1) can be rewritten and expressed in the form of unrestricted ECM for a two-variable case, as shown in Equation (2).


(2) $$\begin{array}{l} \Delta y_{t-1}=c{+\delta }_{1}y_{t-1}+\delta_{2}x_{t-1}+\sum_{i=1}^{p-1}\lambda_{1}\Delta y_{t-i}\\ \;+\sum_{i=1}^{p-1}\lambda_{2}\Delta x_{t-i}+e_{t} \end{array}$$

where c is a constant; Δ is the operator for forwarding difference; *p* indicates the lag order; *i* represents the lag index; *t* stands for the periods; *e*_*t*_ is the disturbance term; *x* is the independent variable; *y* denotes the dependent variable; λ_1_, λ_2_, δ_1_, *and δ*_2_ are coefficients of the lagged variables. In this study, we denote globalization as the independent variable and CO_2_ emissions as the dependent variable.

As shown in Pesaran et al. (49), if the null hypotheses below are rejected following an F-test and *t*-test, a co-integration relationship exists between the variables:

1. *H*_0*A*_ : δ_1_ = δ_2_ = 0
2. *H*_0*B*_ : δ_1_ = 0

To obtain more information about the cointegration status of the variables, McNown et al. (50) propose an additional *t*-test (t_indev) to test the *H*_*oc*_ null hypothesis:

3. *H*_0*C*_ : δ_2_ = 0

McNown et al. (50) identify two degenerate cases which meet the following requirements: (1) *H*_0*A*_ *and H*_0*C*_ *are rejected* while *H*_0*B*_ *is not rejected* and (2) *H*_0*A*_ *and H*_0*B*_ *are rejected* while *H*_0*C*_ *is not rejected*. They further tabulate critical values for the tests using the bootstrap simulation, which rules out possible indecisive cases based on Pesaran et al.'s (49) chart of critical values. According to McNown et al. (50), the bootstrap ARDL test outperforms the asymptotic test with respect to statistical power and size.

To deal with structural breaks in time series, several studies [e.g., (41, 51)] incorporate dummy variables into their models. However, instead of dummy variables, the Fourier approximation can be used to capture both moderate and sharp breaks in time series which have an unknown number of breaks a priori (52, 53). For instance, Yilanci et al. (39) incorporate the Fourier approximation into the bootstrap ARDL model to capture unknown structural breaks when examining the cointegration status of the variables. Thus, a Fourier function is inserted to capture structural breaks, and is formulated as follows:


(3) $$\begin{array}{l} d\left(t\right)=\overset{n}{\underset{k=1}{\sum }}\alpha_{k}sin\left(\frac{2\pi kt}{T}\right)+\overset{n}{\underset{k=1}{\sum }}\beta_{k}cos\left(\frac{2\pi kt}{T}\right) \end{array}$$

where T represents the sample size; *n* is the number of frequencies; π is a constant (3.14159); *t* represents the trend, and *k* is the specific single frequency. Ludlow and Enders (54) suggest that the approximation uses one type of frequency. Hence, the Fourier function is redefined as follows:


(4) $$\begin{array}{l} d\left(t\right)=\gamma_{1}sin\left(\frac{2\pi kt}{T}\right)+\gamma_{2}cos\left(\frac{2\pi kt}{T}\right) \end{array}$$

The Fourier function is subsequently incorporated into Equation (2); the complete model for which is formulated as follows:


(5) $$\begin{array}{l} \Delta y_{t}=c+\gamma_{1}sin\left(\frac{2\pi kt}{T}\right)+\gamma_{2}cos\left(\frac{2\pi kt}{T}\right)+\delta_{1}y_{t-1}+\delta_{2}x_{t-1}\\ \;+\sum_{i=1}^{p-1}\lambda_{1}\Delta y_{t-i}+\sum_{i=1}^{p-1}\lambda_{2}\Delta x_{t-i}+e_{t} \end{array}$$

This aligns with Christopoulos and Leon-Ledesma (55) who use integer frequencies to capture temporary breaks and fractional frequencies to catch permanent breaks. We also allow k in Equation (5) to take fractional values to capture the permanent effect of the structural changes. The optimal value of k is determined when the sum of squared residuals is minimized by exploring all possible frequencies in increments of 0.1 from 0.1 to 5.

## Results and discussion

### The unit root tests

Three common procedures for testing unit root tests, KPSS, ADF, and PP, were implemented to test the variables for stationarity. The null hypothesis states that a time series has a unit root. If the null hypothesis is rejected, the time series is stationary. If not rejected, it is not stationary. In Table 2, the results indicate that CO_2_ emissions in the United States are integrated at I (0) order while all other variables for the three countries are integrated at I (1) order. Thus, all of the variables are stationary at level or at the first difference. McNown et al. (50) suggest that the bootstrap ARDL model is appropriate for testing the co-integration relation between variables if no variables are integrated at I (2) order or above.

**Table 2:** Results of the unit root test.

		**I (0) level**			**I (1) first differences**		
**Country**	**Variables**	**ADF test**	**PP test**	**KPSS test**	**ADF test**	**PP test**	**KPSS test**
China	CO_2_	−0.14 (1)	0.90 (1)	0.85 (0)^***^	−4.29 (1)^***^	−4.29 (1)^***^	0.15 (0)
	Globalization	0.33 (1)	0.49 (1)	0.90 (0)^***^	−5.30 (1)^***^	−5.49 (1)^***^	0.35 (0)
The United States	CO_2_	−2.99 (1)^**^	−2.84 (1)^*^	0.89 (0)^***^	−5.21 (1)^***^	−5.18 (1)^***^	0.47 (0)^**^
	Globalization	−1.66 (1)	−1.68 (1)	0.90 (0)^***^	−6.64 (1)^***^	−6.64 (1)^***^	0.36 (0)^**^
India	CO_2_	−1.25 (1)	−1.33 (1)	0.29 (0)	−8.32 (1)^***^	−8.18 (1)^***^	0.62 (0)^**^
	Globalization	−0.42 (2)	−0.03 (0)	0.85 (0)^***^	−2.62 (1)^*^	−4.59 (1)^***^	0.18 (0)

*** Represent 1% significance level,

** Denote significance in 5%, and

* Represent the levels of significance in 10%. The value in parentheses indicates the optimal lag length of the ADF and PP tests as selected by the Schwert (56) criteria or bandwidth determined by the KPSS test.

### Cointegration test

Table 3 indicates whether there is a Fourier function in the time series model for each country. Rejection of the null hypothesis implies that γ_1_ and γ_2_ equal to zero is rejected for all the countries. In this case, both gradual and sharp breaks are taken into account. The findings provide significant evidence for the existence of a Fourier function in the model (*p*-value < 0.10). The Fourier function is employed to investigate the relationship between globalization and CO_2_ emissions by performing cointegration and causality tests for the United States, China, and India.

**Table 3:** The existence of a Fourier function—China, the United States, and India.

**Country**	**Dep|Ind**	**F**	**Results**
China	CO_2_|Glo	5.322***(0.009)	A Fourier function exists
The United States	CO_2_|Glo	9.022***(0.000)	A Fourier function exists
India	CO_2_|Glo	14.150***(0.000)	A Fourier function exists

The asterisks *** denote the significance at the 1% level. F is the F-statistics for the coefficients of γ_1_ and γ_2_. Additionally, (.) are *p*-value for the coefficients. Dep stands for the dependent variable; Ind stands for the dependent variable.

A bootstrap ARDL model with a Fourier function test is applied to explore the long-term relationship between globalization and CO_2_ emissions for the three CO_2_-emitting counties. It is based on error-correction models without restrictions, where the optimal order of the lags for the variables is determined according to the Akaike and Schwarz information criteria used in other empirical studies (49, 51, 57, 58). The test statistics generated in the bootstrapping process determine whether cointegration between the variables exists. As shown in Table 4, F_a_ is the calculated F test statistic for the coefficients of y_t−1_ and x_t−1_. Additionally, t_dev denotes the *t*-test statistics for the dependent variable, and t_indev denotes the *t*-test statistics for the independent variable. F_a_, t_dev, and F_indev statistics are checked to determine whether the statistical values exceed their corresponding critical values. The null hypothesis of no-cointegration is rejected as all three tests were statistically significant. As shown in Table 3, the values of F_a_, t_dev, and F_indev statistics exceed corresponding critical values for the United States, indicating that globalization and the environment converge in the long term, which is in line with major empirical findings (33, 59–61). However, the results are contrary to the finding in the study of Pata and Yilanci (62) where cointegration does not exist in the United States, the United Kingdom, France, and Germany.

**Table 4:** Cointegration between variables.

**Country**	**Dv|Inv**	**F_a_**	F*	**t_dev**.	t*_dev.	**t_indev**.	t*_indev.	**Conclusions**
China	CO_2_|Glo [3]	7.975^**^	4.980	−3.960^**^	−2.673	−3.89	0.202	Degenerate cases#2
The United States	CO_2_|Glo [1]	13.97^***^	8.011	−4.65^***^	−3.815	4.510^***^	1.494	Cointegration
India	CO_2_|Glo [3]	8.987^*^	8.126	−3.465	−3.794	−4.225	2.213	No cointegration

The number in bracket represents the optimal lag selected by AIC.

***, **, * Represent the levels of significance in 1, 5, and 10%, respectively.

### Granger causality test

Empirical results regarding the impact of globalization on CO_2_ emissions for the three countries are presented in Table 5. In the case of China, the positive causality between globalization and CO_2_ emissions runs from the former to the latter, implying that enhanced globalization leads to environmental degradation. This is in line with major empirical findings (24, 25, 27, 63). The results from a study by Alola and Joshua (63) indicate that globalization increases environmental degradation in upper-middle-income countries. Reasons for the positive causal relationship between globalization and CO_2_ emissions in China include energy use, industrial structure, and unbalanced urbanization. Regarding the United States and India, the negative causality that runs from globalization to CO_2_ emissions implies that accelerated globalization promotes the quality of the environment, which is in line with major empirical findings (7, 26). However, the results are contrary to the finding in the study of Shehzad et al. (33). In the United States, state-of-the-art technology, knowledge of industrial processes, and human capital from around the world help combat pollution and cut CO_2_ emissions. Having initiated the International Solar Alliance, India has begun its journey to net-zero emissions through a close alliance with the European Union aimed at combating climate change (64). The underlying reasons for the negative causal relationship between globalization and CO_2_ emissions in the United States could be the development of renewable energy and awareness of quality of life, and renewable-related investments and the promotion of renewable infrastructure in India.

**Table 5:** The results in granger causality in short term.

**Country**	***ΔCO*_2_ equation:** ***ΔGlo* F-statics,** **(*p*-value) (sign)**
China	6.24^***^(0.001) (+)
U.S.A	−2.97^***^(0.005) (–)
India	4.365534^***^(0.010) (–)

*** Represent the levels of significance in 1%.

## Conclusion

The study revisited the globalization-environment nexus in China, the United States, and India from 1970 to 2018. A bootstrap ARDL with a Fourier function procedure was applied to test cointegration and confirm the presence of a long-term relationship between globalization and the environment. This study enriches current literature by providing new evidence derived from a relatively rigorous method and a comprehensive globalization index to confirm the long- and short-term relationship between globalization and the environment. Such cointegration provides useful insights that will facilitate the creation of effective strategies to maintain sustainable development in the long term. Furthermore, the directions of the causal relationships provide useful insights that will support policymakers in developing better economic and environmental policies to mitigate environmental degradation.

The empirical findings have important implications for policymakers. First, in the context of China, the causal link between the globalization index and CO_2_ emissions indicates that augmenting globalization of the economy increases environmental degradation. One of the causes of such degradation is China's strong reliance on energy use for economic growth. However, China will benefit when its government reforms regulations to establish a robust energy infrastructure and greater efficiency through the implementation of energy conservative policies (59). Globalization also injects more vocational opportunities into the economy and society in urban areas. For example, the transportation infrastructure supports the exchange of resources between regions and increases information transparency in economic and social activities (65). However, regions with more energy-intensive industries cannot dramatically change their industrial structure due to regional differences and the stage characteristics of industrial development (66). Thus, along with unbalanced urbanization, the increasing usage of commercial electricity and other natural resources is also responsible for environmental degradation. If the rising trend toward globalization of economic and social activities continues at the expense of environmental degradation, it might result in increased energy consumption and a loss of natural resources, ultimately impeding sustainable development in China. These results support the work of Yurtkuran (24), who found that globalization plays a significant role in environmental pollution.

Secondly, in the United States and India, the negative causal relationship between the globalization index and CO_2_ emissions indicates that increasing globalization of the economy improves environmental quality. In addition, in the United States, the nexus between globalization and the environment is cointegrated in the long term. These results imply that the process of globalization generates a better economic environment in the United States and India. In recent decades, as the pace of globalization has accelerated, the United States government has become increasingly aware that globalization may have a negative impact on the environment. Hence, related initiatives such as promoting the development and use of renewable energy are being considered. The amount of money invested in renewable energy technologies in the United States has increased dramatically over the previous decade, rising from 11 billion USD in 2005 to over 46 billion USD. In terms of long-term scenarios, the United States is the only country in which the globalization and environment nexus are cointegrated. This suggests these dimensions constitute dynamic adjustments to equilibrium to maintain sustainability in the long term. As a result of globalization, state-of-art technology and knowledge are being applied to businesses, and thus the wealth of citizens is increasing. Concomitantly, awareness of quality of life also increases. The greater the globalization in the economy, the stronger the reinforcement effect between the output from novel technology and knowledge and the degree of awareness regarding environmental development. For India, the findings imply that the economy in a developing country benefits from economic and social activities during globalization and reaches a higher level. The strategies employed by its government for economic and social development strive to support environmentally-friendly activities rather than profit-oriented activities, as the level of domestic income increases under globalization. In recent years, India has supplied almost as much new solar generation capacity as the United States did in 2018 (67). The government has also set a goal of 450 gigawatts of installed renewable energy capacity. The economic goal is to secure more renewable-related investments. In addition, India is one of the largest users of groundwater in the world. Over-extraction of groundwater also results in increased CO_2_ emissions. For farmers to sustain their operations, a program with a renewable infrastructure is required that assists them in working with solar energy rather than traditional forms of energy such as diesel (68).

We recommend that the Chinese government releases restrictions on international trade to accelerate market integration in the global supply chain. This will enhance economic wealth and promote an awareness of environmental development. The United States government should continue removing economic and social barriers affecting its regional and global partners. Also, it should be concerned about environmental development when accelerating globalization through economic and social activities. The Indian government should continue investing in renewable energy as energy-intensive industries are the main source of CO_2_ emissions in India. In sum, policy implementations by governments and the availability of financial tools are essential, as economies will gain from globalization through interconnected state-of-the-art technology and knowledge. However, a limitation of this research is that it focuses on the nexus of globalization and the environment in specific nations. A panel study is therefore recommended to further validate this nexus.

## Data availability statement

Publicly available datasets were analyzed in this study. This data can be found here: https://data.worldbank.org/.

## Author contributions

C-FW contributed to the research topic, research model, statistical analysis, and writing. TC contributed to the research topic and research model. T-PW and K-jL contributed to the data collection. M-CL and S-CH contributed to the statistical analysis and writing. All authors contributed to the article and approved the submitted version.

## Funding

This study were supported by Hubei University of Economics with the grant number of XJ201901, XJ201902, and 11024225 and Research Center of Hubei Logistics Development.

## Conflict of interest

The authors declare that the research was conducted in the absence of any commercial or financial relationships that could be construed as a potential conflict of interest.

## Publisher's note

All claims expressed in this article are solely those of the authors and do not necessarily represent those of their affiliated organizations, or those of the publisher, the editors and the reviewers. Any product that may be evaluated in this article, or claim that may be made by its manufacturer, is not guaranteed or endorsed by the publisher.
